# Are Algae a Good Source of Antioxidants? Mechanistic Insights into Antiradical Activity of Eckol

**DOI:** 10.3390/ijms26189223

**Published:** 2025-09-21

**Authors:** Maciej Spiegel

**Affiliations:** Department of Organic Chemistry and Pharmaceutical Technology, Faculty of Pharmacy, Wroclaw Medical University, Borowska 211A, 50-556 Wroclaw, Poland; maciej.spiegel@umw.edu.pl

**Keywords:** eckol, antioxidant, hydrogen atom transfer, density functional theory, alga, molecular modeling, radical scavenging, polyphenol, oxidative stress, reactive oxygen species

## Abstract

Eckol (**Eck**), a polyphenolic compound of marine origin, exhibits strong scavenging activity against hydroperoxyl radicals. This study explores its acid-base speciation in aqueous media and evaluates its antioxidant potential through electronic, thermochemical, and kinetic analyses under biologically relevant conditions. The deprotonated species of **Eck** display exceptionally high rate constants for hydrogen atom transfer, indicating a potent antiradical mechanism. The apparent rate constant, accounting for species distribution at physiological pH and the molar fraction of ^•^OOH, was calculated as 1.09 × 10^7^ M^−1^·s^−1^. Compared to related compounds, **Eck** demonstrates outstanding hydroperoxyl radical-scavenging capacity, supporting its potential as a natural antioxidant in biological systems.

## 1. Introduction

Polyphenols represent a diverse group of naturally occurring chemical compounds that have attracted substantial attention across the chemical, biological, and medical sciences. Their widespread application spans from food preservation to pharmaceutical formulations, primarily due to their potent antioxidant properties. Biologically, antioxidants—especially those derived from natural sources—play a crucial role in maintaining the physiological levels of reactive oxygen species (ROS), thereby preventing the oxidative damage that occurs when these species accumulate excessively [1].

ROS such as hydroperoxyl and hydroxyl radicals are highly reactive and capable of transferring energy to essential biomolecules, including lipids, proteins, and nucleic acids. This leads to a cascade of molecular damage, ultimately impairing cellular function and contributing to the pathogenesis of various diseases. Neurodegenerative disorders provide notable examples: Parkinson’s disease is associated with the oxidative destruction of dopaminergic neurons [2], while Alzheimer’s disease involves the aggregation of oxidized amyloid plaques [3]. Beyond neurological conditions, oxidative stress has also been implicated in cardiovascular [4,5,6], metabolic [4,7], and respiratory disorders [8,9]—collectively recognized as major non-communicable diseases of the 21st century. Given this context, the sustained intake of antioxidants has been consistently associated with improved health outcomes and disease prevention [10].

At the molecular level, plant antioxidants neutralize ROS primarily by donating electrons or hydrogen atoms, converting themselves into relatively stable radical species in the process [11]. This transformation is rendered energetically favorable due to the delocalization of unpaired electron density over the aromatic structures of polyphenols. Three structural features are widely recognized as critical to their antioxidant capacity: (1) the presence of hydroxyl groups, which serve as hydrogen donors; (2) the aromatic rings, which facilitate resonance stabilization of the resulting radicals; and (3) the positional arrangement of these hydroxyl groups and other functional motifs, which modulate the efficiency of radical scavenging.

**Figure 1:**
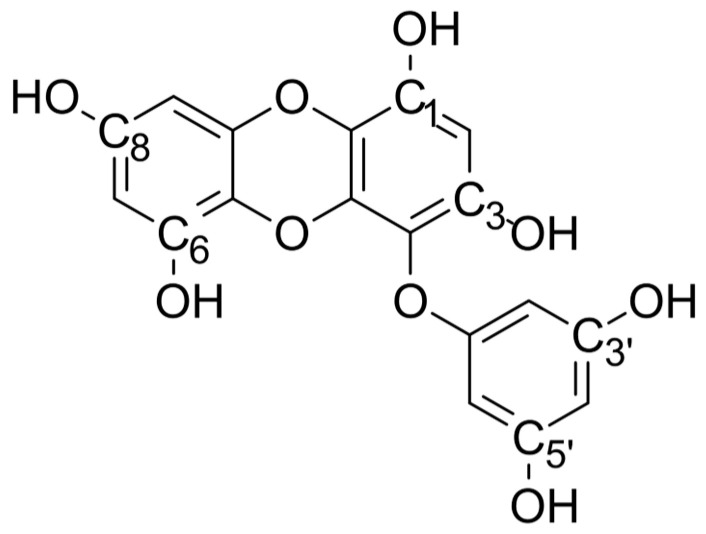
Structure of Eckol.

Phlorotannins in brown algae are highly heterogeneous, including simple monomers (phloroglucinol), low-molecular-weight oligomers like Eckol (**Eck**, Figure 1), and much larger polymers of up to ~100–650 kDa [12,13]. Polymeric phlorotannins have been linked not only to antioxidant defense but also to UV protection [14], herbivore deterrence [15], and the structural reinforcement of algal cell walls [13], suggesting multifunctionality beyond radical scavenging. **Eck** is particularly abundant in the genus *Ecklonia* (e.g., *E. cava*, *E. stolonifera*, *E. maxima*) and in *Eisenia bicyclis* or *Ishige okamurae* [16,17,18], found in coastal marine environments in East Asia (Japan, Korea). This substance has shown promising biological activities, including antiplasmin inhibition, radioprotective effects, cytoprotection against oxidative stress, and antithrombotic and profibrinolytic properties [18]. Given that, to date, **Eck** has been isolated from natural sources and the yields of **Eck** vary depending on extraction and seasonal factors [19]—for instance, pure **Eck** was obtained from *E. cava* after methanol extraction followed by HPLC purification, yielding ~20 mg per 1 kg dry alga [20]—little is known about its actual antioxidative potential, and, in general, the mechanistic behavior of marine-derived phlorotannins remains poorly characterized.

To fill this gap, a combined thermochemical and kinetic approach—an advanced and well-established methodology in antioxidant research—was employed to probe the radical-scavenging potential of **Eck** against hydroperoxyl radicals (^•^OOH). This study represents the very first such systematic computational evaluation of **Eck**, explicitly considering acid–base speciation and calculating diffusion-limited rate constants under physiological pH. The findings provide a deeper understanding of the molecular mechanisms underlying **Eck**’s antioxidant activity, offer insights into its potential applications, and lay the groundwork for future studies exploring marine phlorotannins as efficient antioxidants.

## 2. Results and Discussion

### 2.1. Eckol Speciation in Water

**Figure 2:**
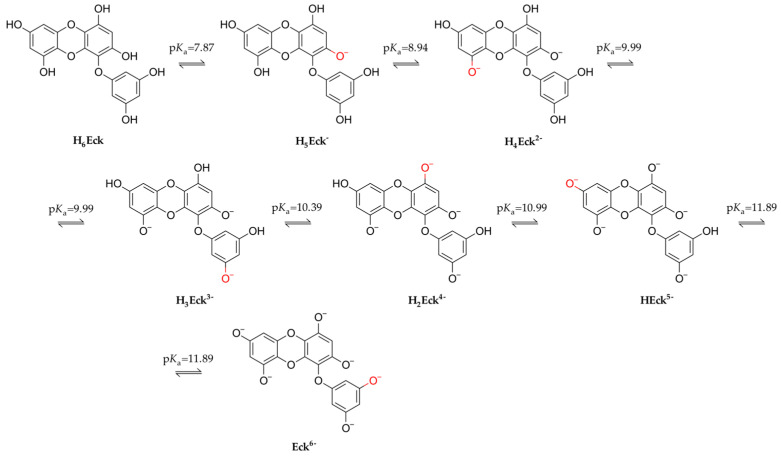
Deprotonation pathway and corresponding dissociation constants for each step.

The different acid–base species of **Eck** can significantly influence its observed biological activity. To ensure that this study focuses on the biologically relevant forms of **Eck**, its dissociation constants and molar fractions at physiological pH were assessed using a fitted-parameters method [21] (Figure 2). In the absence of experimental data, these theoretical predictions were considered reliable due to the validated nature of the approach employed. It ensures that the studies forms correspond to the species actually present in human physiological environments, rather than hypothetical or non-relevant protonation states.

Deprotonation begins at a relatively high pH of 7.87 and involves the hydroxyl group at C_3_, located adjacent to the phloroglucinol unit. Subsequent deprotonation steps involve the hydroxyl groups at C_6_, C_5′_, C_1_, C_8_, and finally C_3′_, with p*K*_a_ values of 8.94, 9.99, 10.39, 10.99, and 11.89, respectively. This results in a solution predominantly composed of the neutral species (**H_6_Eck**, 74.33%), followed by the monoanion (**H_5_Eck^−^**, 24.95%), and a minor fraction of the dianion, (**H_4_Eck^2−^**, 0.71%).

### 2.2. Reactivity Indices

The antioxidant activity of polyphenolic compounds through formal hydrogen atom transfer (*f*-HAT, Equation (1)), in which the polyphenolic OH donates a hydrogen atom to a radical, and single-electron transfer (SET, Equation (2)), in which an electron is transferred from the polyphenol to the radical. These mechanisms can be preliminarily evaluated using two key reactivity indices: bond dissociation energy (BDE, Equation (3)) and adiabatic ionization potential (aIP, Equation (4)), respectively. The lower the value of each index, the more favorable the corresponding mechanism is expected to be. While not universally predictive, previous studies have shown that the Bell–Evans–Polanyi [22,23] principle often applies to antiradical reactions [24], supporting the validity of this approach.

H–A + R^•^ → A^•^ + R–H (1)

HA + R^•^ → HA^•+^ + R (2)

BDE = E(A^•^) + E(H^•^) − E(HA) (3)

aIP = E(HA^•+^) + E(e^–^) − E(HA) (4)

**Figure 3:**
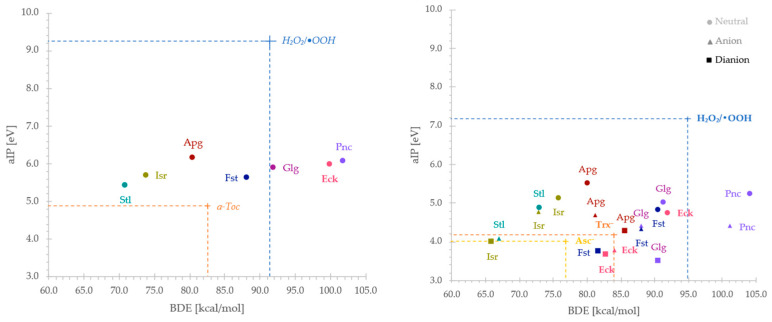
Electron–hydrogen-donating ability map (eH-DAMA) for pentyl ethanoate (**left**) and water (**right**), constructed using BDE and aIP values for eckol and reference compounds (Apg—apigenin; Isr—isorhamnetin; Stl—scutellarein; *a*-Toc—ɑ-Tocopherol; Fst—fisetin; Glg—galangin; Pnc—pinocembrin; Asc^−^—ascorbate; Trx^−^—trolox monoanion) [26,27,28,29,30,31].

A useful method for visualizing and comparing antioxidant properties across different compounds—including reference substances—is the electron–hydrogen-donating ability map (eH-DAMA) [25], presented here for both pentyl ethanoate and aqueous environments (Figure 3). Despite a common protocol for evaluating antiradical activity is followed, individual studies often readjust it. In this context, it is essential to compare results that are consistent with the methodology in order to derive meaningful insights. Therefore, only previously investigated compounds—apigenin (**Apg**) [26], isorhamnetin (**Isr**) [27], galangin (**Glg**) [28], pinocembrin (**Pnc**) [29], fisetin (**Fst**) [30], and scutellarein (**Stl**) [31]—were considered.

As shown, **H_6_Eck** in pentyl ethanoate (**H_6_Eck^PET^**) exhibits an aIP of 6.0 eV, comparable to that of **Glg** (5.9 eV) [28] and **Pnc** (6.1 eV) [29], both flavonoids commonly found in honey. The lowest BDE value observed (99.9 kcal·mol^−1^) is relatively high in absolute terms, suggesting that while **H_6_Eck^PET^** may be a slightly better radical scavenger by *f*-HAT than **Pnc** (101.9 kcal·mol^−1^), its antioxidant effectiveness through hydrogen donation remains limited. As a result, **H_6_Eck^PET^** may primarily scavenge ^•^OOH with electron donation rather than hydrogen transfer; however, it does not outperform α-tocopherol (**α-Toc**) in this respect.

In contrast, the aqueous environment significantly alters the reactivity profile. Even in its undissociated form, **H_6_Eck** shows the potential to scavenge ^•^OOH by both mechanisms. While its BDE is slightly lower than **Pnc** (92.0 vs. 104.1 kcal·mol^−1^), the most notable change is the substantial drop in aIP to 4.7 eV, the lowest among the set of neutral species analyzed. Further deprotonation induces a dramatic shift in redox properties: in its monoanionic form, **Eck** exhibits a sharp reduction in both BDE (84.1 kcal·mol^−1^) and aIP (3.8 eV). This enhanced electron-donating ability allows **H_5_Eck^−^** to outperform not only Trolox (**Trx^−^**), a strong biological reductant, but potentially any other anionic flavonoid studied. **H_4_Eck^2−^** shows a further, albeit less pronounced, decrease in reactivity indices, with BDE and aIP values of 82.9 kcal·mol^−1^ and 3.6 eV, respectively.

### 2.3. Thermochemistry and Kinetics of Type I Antioxidative Reactivity

The thermochemical parameters associated with **Eck**’s scavenging activity against ^•^OOH are presented in Table 1 (for pentyl ethanoate) and Table 2 (for water).

In pentyl ethanoate, **H_6_Eck^PET^** exhibits weak antioxidative performance. As computed, only one reaction pathway—*f*-HAT from the C_1_ hydroxyl—is marginally favorable according to the energetic threshold of 10 kcal·mol^−1^ imposed by the evaluation protocol, with a ΔG of 9.2 kcal·mol^−1^ and a low rate constant of 2.25 × 10^−4^ M^−1^·s^−1^. All other hydroxyls and RAF pathways remain energetically inaccessible, highlighting the limited reactivity in lipid-like environments.

In aqueous solution, however, the true antioxidant potential of **Eck** becomes apparent. **H_6_Eck** is a modest scavenger—it displays the exergonic character of *f*-HAT from C_1_, C_3_, C_6_, and C_8_ hydroxyls, with rate constants on the order of ×10^4^. The deprotonated forms, **H_5_Eck^−^** and **H_4_Eck^2−^**, demonstrate even higher reactivity, undergoing barrierless, diffusion-limited *f*-HAT reactions from multiple hydroxyl groups—these processes show no activation energy, as confirmed by the monotonically decreasing PES curve along the hydrogen transfer coordinate. For instance, the barrierless reactions for **H_5_Eck^−^** occur at C_1_, C_3′_, C_5′_,and C_6_ hydroxyls, indicating these as the most potent sites for hydrogen donation in the monoanionic form. Similarly, **H_4_Eck^2−^** shows barrierless *f*-HAT at C_1_, C_3′_, C_5′_, and C_8_. It can be concluded that C_1_ is a key contributor to the substance’s antiradical activity, being consistently linked with a reasonable branching ratio. This enhanced activity in the anionic forms underscores the role of deprotonation in lowering energy barriers and facilitating rapid hydrogen abstraction, likely due to its increased electron density and better solvation in water.

While radical adduct formation pathways are generally much slower than *f*-HAT across all examined species, they still offer insights into the spin delocalization and bonding preferences of the phenolic moiety. For instance, accessible RAF pathways in **H_5_Eck^−^** and **H_4_Eck^2−^**, such as at C_4b_ and C_8b_, have rate constants orders of magnitude lower than *f*-HAT, suggesting that most of the radical stabilization occurs at aromatic hydroxyls rather than at carbon centers. Simultaneously, the propensity for SET increases upon deprotonation, consistent with prior findings for polyphenolic compounds [32,33]. This trend validates the predictions made by the eH-DAMA map in Section 2.2.

Overall, the collected data reveal a stark solvent-dependent behavior: **Eck**’s antioxidative efficacy is minimal in non-polar media but rivals or exceeds that of potent natural antioxidants in aqueous environments, particularly in its deprotonated states, with total rate constants reaching up to 1.91 × 10^10^ M^−1^·s^−1^ for **H_4_Eck^2−^**.

Although the individual values are remarkably high, the observable antioxidant activity in solution is governed by the relative abundance of each species at a given pH. Therefore, the apparent rate constant (*k_app_*), representing the effective scavenging ability of **Eck** in water, was calculated as a weighted sum of the individual rate constants (*k_i_*), adjusted by their molar fractions (*x_i_*) and the molar fraction of ^•^OOH at pH = 7.4 (0.0025), as expressed in Equation (5):

(5) $$k_{app}=0.0025\sum_{i}x_{i}\times k_{i}$$

The resulting value equals 1.09 × 10^7^ M^−1^·s^−1^. When compared with flavonoids, **Eck** fits into the following reactivity trend: Pnc < Apg < Stl < Glg < Fst < **Eck** < Isr. This shows that **Eck** is competitive with, or even outperforms, many well-known terrestrial antioxidants under comparable aqueous conditions, highlighting its exceptionally high antiradical potential.

Theoretical research across phlorotannins is still rare. Only recently has diphlorethol been examined with a computational approach, where it displayed slightly higher activity than **Eck** in water [34]. These results suggest that small differences in structural arrangement within low-molecular-weight phlorotannins can significantly alter radical-scavenging efficiency. The topic is to be studied more extensively in the future.

## 3. Materials and Methods

The study was conducted using quantum mechanical methods, as implemented in the Gaussian 16 (Rev. C.02) software package [35]. QM calculations are particularly useful for antioxidant studies because they allow one to quantify the energy required for key processes, such as hydrogen atom transfer or single-electron transfer, which underlie radical scavenging.

To ensure that all relevant three-dimensional shapes (conformers) of **Eck** were considered, a conformational sampling using CREST was performed with metadynamics algorithms [36,37]. This approach systematically explores the potential energy surface and provides a set of low-energy conformers. The most stable conformers were then optimized at the M06-2X/6-311+G(d,p) level of theory [38,39,40,41,42,43], a density functional method that has been validated for thermochemistry and kinetics of antioxidant reactions. Optimizations were performed in two solvent environments—pentyl ethanoate (the largest lipid-phase solvent available in the software, with a dielectric constant of ~4.7, comparable to biological membranes, and validated in prior QM-ORSA studies as a representative lipid-phase model) and water—using the SMD implicit solvation model [44]. This setup allowed to approximate both biological membranes and aqueous intracellular conditions. All thermochemical and kinetic computations were carried out at 298.15 K (25 °C). For the aqueous solution, speciation and rate-constant calculations assumed pH 7.4, corresponding to mammalian physiological conditions.

At each stage of optimization, the ground-state character of the structures was confirmed by frequency calculations, which yielded no imaginary frequencies. Transition-state (TS) optimizations were performed to locate the intermediate geometries between reactants and products. The identity of each TS was confirmed by intrinsic reaction coordinate (IRC) calculations, which ensure that the TS connected the correct reactants and products [45].

Reaction rate constants were derived from activation free energies using Transition State Theory, which links the probability of barrier-crossing to kinetic rates. For SET processes, activation energies were obtained using Marcus theory [46,47,48], which accounts for solvent reorganization during electron transfer. These approaches follow the QM-ORSA protocol [11,49,50,51], which has been widely applied to predict antioxidant activity in polyphenols [11,26,27,28,29,30,31,50,52].

## 4. Conclusions

This work demonstrates that the antioxidative behavior of **Eck** is highly dependent on its protonation state, with anionic species showing barrierless and diffusion-limited reactivity toward hydroperoxyl radicals. The remarkably high rate constants for certain of indivial species, reaching ~10^10^ M^−1^·s^−1^, indicate that **Eck**’s radical-scavenging capacity is not uniform but is governed by its acid–base equilibrium at physiological pH. 

Nonetheless, while the computational results show plausible activity for **Eck**, in vivo, most phlorotannins are present as higher-degree oligomers or polymers rather than as free monomers [53]. They may exhibit reduced mobility, steric hindrance, and limited access to short-lived reactive oxygen species. As a result, **Eck**’s effectiveness once incorporated into oligomeric or polymeric assemblies may be attenuated. At the same time, larger phlorotannins carry a higher density of phenolic groups, and extended conjugation may actually enhance radical stabilization. To clarify these aspects, future computational studies should extend to dimeric and trimeric structures (e.g., dieckol and bieckol), and ideally to representative polymer fragments, to assess whether they retain the fast hydrogen atom transfer reactivity observed for **Eck** or whether polymerization entails a systematic trade-off. Such efforts would provide a more realistic picture of how low-molecular-weight units contribute to the overall protective chemistry of brown algae. 

Simultaneously, experimental validation is anticipated to enable a more direct comparison between the theoretical predictions and actual antioxidant activity.

## Figures and Tables

**Table 1:** Gibbs free energies of reaction (ΔG, in kcal·mol^−1^) ^1^ for ^•^OOH scavenging in pentyl ethanoate. Activation energies (ΔG^≠^, in kcal·mol^−1^) ^1^, rate constants (*k*, in M^−1^·s^−1^) ^2^, and branching ratios (Γ, in %) are tabulated for accessible pathways only.

Mechanism	H_6_Eck^PET^			
	ΔG	ΔG^≠^	*k*	Γ
*f*-HAT				
C_1_	9.2	25.2	2.25 × 10^−4^	100.0
C_3_	10.6			
C_3′_	49.6			
C_5′_	16.2			
C_6_	12.9			
C_8_	11.0			
RAF				
C_1_	25.6			
C_1′_	27.9			
C_2_	27.8			
C_2′_	28.7			
C_3_	25.2			
C_3′_	31.5			
C_4_	26.5			
C_4a_	24.2			
C_4b_	22.5			
C_4′_	28.7			
C_5_	28.8			
C_5′_	31.5			
C_6_	28.8			
C_6′_	28.9			
C_7_	27.1			
C_8_	61.8			
C_8a_	26.0			
C_8b_	22.8			

^1^ Gibbs free energies and activation energies are calculated at the 1 M standard state and include solvent cage effects. ^2^ Rate constants include Eckart tunneling corrections and account for diffusion limits.

**Table 2:** Gibbs free energies of reaction (ΔG, in kcal·mol^−1^) ^1^ for ^•^OOH scavenging in water. Activation energies (ΔG^≠^, in kcal·mol^−1^) ^1^, rate constants (k, in M^−1^·s^−1^) ^2^, and branching ratios (Γ, in %) are tabulated for accessible pathways only.

\	H_6_Eck				H_5_Eck^−^				H_4_Eck^2−^			
	ΔG	ΔG^≠^	*k*	Γ	ΔG	ΔG^≠^	*k*	Γ	ΔG	ΔG^≠^	*k*	Γ
*f*-HAT												
C_1_	−3.5	15.3	5.39 × 10^4^	26.12	−11.4	0.0 ^3^	4.23×10^9^	24.98	−13.4	0.0 ^3^	4.24×10^9^	22.21
C_3_	−4.4	15.4	6.08 × 10^4^	29.47								
C_3′_	17.7				−2.0	0.0 ^3^	4.23 × 10^9^	24.98	−4.3	0.0 ^3^	4.24 × 10^9^	22.21
C_5′_	17.7				−2.2	0.0 ^3^	4.23 × 10^9^	24.98	−4.7	0.0 ^3^	4.24 × 10^9^	22.21
C_6_	−2.4	16.4	5.68 × 10^4^	27.52	−2.4	0.0 ^3^	4.23 × 10^9^	24.98				
C_8_	−2.2	16.0	3.17 × 10^4^	15.36	−2.6	17.7	6.36 × 10^3^	0.00	−10.3	0.0 ^3^	4.24 × 10^9^	22.21
RAF												
C_1_	11.9				12.4				10.7			
C_1′_	12.3				15.2				15.1			
C_2_	13.0				9.0	13.9	4.26 × 10^2^	0.00	8.8	13.5	2.66 × 10^3^	0.00
C_2′_	12.9				13.5				13.3			
C_3_	10.0				11.9				11.4			
C_3′_	15.0				15.5				14.7			
C_4_	10.4				1.9	11.1	5.01 × 10^4^	0.00	1.5	10.2	1.63 × 10^6^	0.01
C_4a_	8.9	19.0	1.03 × 10^−1^	0.00	7.1	15.7	1.91 × 10^1^	0.00	6.4	12.2	6.56 × 10^4^	0.00
C_4b_	7.0	12.8	3.12 × 10^3^	1.51	7.2	13.3	1.26 × 10^3^	0.00	1.9	6.2	8.88 × 10^8^	4.65
C_4′_	13.6				14.0				14.6			
C_5_	12.5				12.1				12.6			
C_5′_	15.6				15.2				15.7			
C_6_	14.3				13.8				11.3			
C_6′_	15.0				14.8				15.0			
C_7_	12.0				11.8				9.8	16.9	4.10 × 10^1^	0.00
C_8_	44.0				40.6				33.9			
C_8a_	9.6	19.2	7.90 × 10^−2^	0.00	9.9	19.8	2.77 × 10^2^	0.00	8.2	15.8	3.07 × 10^2^	0.00
C_8b_	7.0	15.7	2.22 × 10^1^	0.01	−1.4	7.8	1.35 × 10^7^	0.08	−0.3	6.2	1.23 × 10^9^	6.43
SET												
	26.0	22.3	3.66 × 10^−7^	0.00	4.8	9.4	7.39 × 10^5^	0.00	1.4	7.8	1.17 × 10^7^	0.06
*k*_i_			2.06 × 10^5^				1.69 × 10^10^				1.91 × 10^10^	

^1^ Gibbs free energies and activation energies are calculated at the 1 M standard state and include solvent cage effects. ^2^ Rate constants include Eckart tunneling corrections and account for diffusion limits. ^3^ The reaction was found to be barrierless, as confirmed by a descending potential energy surface (PES) along the hydrogen transfer coordinate from the hydroxyl group to the radical.

## Data Availability

The data will be made available upon reasonable request.
